# Household Surveillance for Norovirus Gastroenteritis in a Nicaraguan Birth Cohort: A Nested Case—Control Analysis of Norovirus Risk Factors

**DOI:** 10.3390/pathogens12030505

**Published:** 2023-03-22

**Authors:** Nadja Alexandra Vielot, Omar Zepeda, Yaoska Reyes, Fredman González, Jan Vinjé, Sylvia Becker-Dreps, Filemón Bucardo

**Affiliations:** 1 Department of Family Medicine, University of North Carolina at Chapel Hill, Chapel Hill, NC 27599, USA; sbd@email.unc.edu; 2 Department of Microbiology, National Autonomous University of Nicaragua, Leon 21000, Nicaragua; 3 Division of Viral Diseases, Centers for Disease Control and Prevention, Atlanta, GA 30329, USA; 4 Department of Epidemiology, University of North Carolina at Chapel Hill, Chapel Hill, NC 27599, USA

**Keywords:** norovirus, acute gastroenteritis, infants, Nicaragua

## Abstract

Norovirus causes a large proportion of pediatric acute gastroenteritis (AGE) worldwide, and no vaccines are currently available. To inform public health measures against norovirus gastroenteritis, we assessed risk factors in a case–control study nested in a birth cohort study in Nicaragua. Between June 2017 and January 2022, we followed children weekly for AGE episodes, and collected stool specimens from symptomatic children. Risk factors for AGE were collected during routine weekly visits. Norovirus was detected in stools using real-time reverse transcriptase polymerase chain reaction and positive specimens were genotyped using Sanger sequencing. We included 40 norovirus-positive AGE children matched 1:2 to controls and conducted bivariate and multivariable analyses of norovirus AGE risk factors. Among typeable norovirus infections, GII.4 were more severe than non-GII.4 (four/twenty-one vs. one/nine) and accounted for all emergency visits and hospitalizations. Adjusted conditional logistic regression found that female sex and higher length-for-age Z score were protective against norovirus AGE; a dirt floor in the home, sharing cups or bottles, and recent contact with someone with AGE symptoms were associated with norovirus AGE, though estimates were highly imprecise. Reducing contact with symptomatic persons and with saliva or other bodily fluids on cups or floors could reduce infant norovirus incidence.

## 1. Introduction

Globally, norovirus is detected in an estimated 18% of cases of acute gastroenteritis (AGE) [1] and causes approximately 214,000 deaths each year [2]. While norovirus affects all age groups, children and older adults bear the highest burden of disease. In Nicaragua, as in many other settings where rotavirus vaccines have been introduced, norovirus replaced rotavirus as the most common enteric pathogen detected in children with AGE [3,4].

Pediatric norovirus vaccines are currently in development, though efforts are hampered by the wide genetic diversity of noroviruses and apparent lack of cross-protection across genotypes [5]. Several vaccine candidates have been evaluated in clinical trials, but absent licensed norovirus vaccines, prevention of norovirus transmission includes common-sense measures, such as hand hygiene, environmental disinfection, and limiting contact with infected persons. As norovirus can be transmitted through contaminated food and water [6,7], improved food safety would also likely decrease norovirus burden. Except for these general measures recommended for the prevention of all fecal–oral pathogens, there is limited scientific evidence on specific modifiable risk factors for norovirus to guide prevention efforts, particularly in young children in low- and middle-income countries (LMIC), who bear a high burden of disease.

Prior studies from LMIC using cross-sectional, cohort, and case–control designs have reported that younger age, larger households, household sanitation and socioeconomic factors, eating outside the household, and having contact with another household member with AGE symptoms were risks for norovirus AGE, while studies were mixed on the benefits of breastfeeding in reducing norovirus AGE [8,9,10,11,12]. The goal of this study was to identify modifiable risk factors for norovirus AGE in a population of young Nicaraguan children, using a larger sample of symptomatic norovirus-infected children than in previous studies and assessing previously unstudied risk factors. Furthermore, to inform vaccine development for norovirus prevention, we compared clinical symptoms and severity in children infected with the globally prevalent GII.4 genotype as compared with other norovirus genotypes. The results of this study add to the available data on non-vaccine interventions to reduce norovirus AGE burden in this setting.

## 2. Materials and Methods

### 2.1. Study Sample and Data Collection

We leveraged the Sapovirus-Associated GastroEnteritis (SAGE) cohort, described elsewhere [13], to perform a nested case–control study of infant norovirus incidence in an urban coastal region of Nicaragua (León). Briefly, we recruited 444 newborns between June 2017 and July 2018 and completed weekly household visits over 36 months to determine incidence of AGE, defined as diarrhea and/or vomiting. A secondary cohort of 80 children was recruited between January and April 2021, and completed 18 months of weekly visits following the same study procedures as the primary cohort, with the exception of providing weekly routine stool specimens. 

At enrollment, we collected data on the child’s birth characteristics, household sanitation, and household socioeconomic status. At weekly intervals, we collected data on AGE symptoms in the past seven days, and breastfeeding and supplementary feeding in the last week. At monthly intervals, we collected additional data on AGE risk factors, including nutritional factors, hygiene practices, and interpersonal contacts, and infant weight and length.

### 2.2. Specimen Collection and Processing

Stools from infants in the secondary cohort experiencing AGE symptoms were collected within 10 days of onset and analyzed for the presence of norovirus using reverse transcriptase quantitative polymerase chain reaction (RT-qPCR) [14]. Parents of the children with norovirus AGE (cases) from the primary and secondary cohorts were invited to participate in a study of norovirus household transmission until 40 cases of infant norovirus infection were recruited; the same 40 cases were leveraged for this case–control analysis. 

Norovirus-positive specimens with Ct values <33 were selected for genotyping; in brief, a segment in the ORF1/ORF2 overlap region was amplified using primers for genogroup (G) I ((Mon432/GISKR) and GII (Mon431/G2SKR) in the RT-PCR assays recommended by Centers of Disease Control and Prevention (CDC) protocols. The PCR products of these reactions (579 bp for GI and 570 bp for GII) were sequenced in the forward and reverse directions by Sanger sequencing [14,15]. Sequences were genotyped using a human calicivirus typing tool [16]. Low viral load (Ct value > 33) precluded successful genotyping in 10 samples. The diagnostic methods were consistent across children and for the duration of the study, whereas genotyping was performed on all samples at once at the conclusion of the data collection period.

### 2.3. Control Selection and Data Analysis

When norovirus was detected in a stool sample, two controls from the cohort were selected with replacement for each case using a risk-set sampling approach. That is, a control could be selected more than once, and children enrolled in the cohort who had not yet experienced a symptomatic norovirus AGE infection at the time the case was diagnosed were eligible to be selected as controls. As such, a child who was selected as a control had the potential to become a norovirus case later in the study. Children who were cases at any point were not eligible to be selected as controls thereafter.

Clinical presentation of norovirus AGE episodes was summarized using descriptive statistics, stratified by GII.4 episodes, other non-GII.4 episodes, and episodes in which the norovirus genotype was not able to be detected (i.e., “untypeable”). AGE severity scores were adapted from the scale developed by Lee et al. [17]. We then evaluated bivariate associations between norovirus case status (case versus control) and known or suspected risk factors for norovirus infection using the chi-square test. Case-control data were analyzed using a multivariable conditional logistic regression model, adjusting for risk factors independently associated with norovirus AGE at α < 0.1 and residual age differences between cases and controls. In contrast to baseline risk factors, risk factors that were collected repeatedly over time (e.g., nutritional practices and interpersonal contacts) were determined using data reported during the most recent monthly survey prior to the norovirus AGE episode, and for the corresponding monthly survey for controls. Thus, we present odds ratios (OR) and 95% confidence intervals (CI) for the association of various risk factors with norovirus case status. Data analyses were performed in SAS version 9.4 (SAS Institute, Research Triangle Park, North Carolina).

## 3. Results

**Figure 1:**
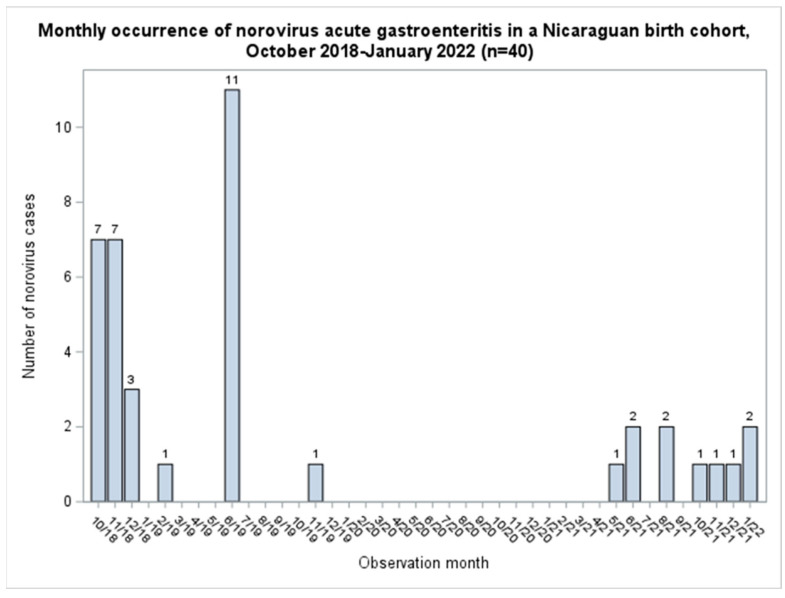
Monthly occurrence of norovirus acute gastroenteritis in a Nicaraguan birth cohort, October 2018—January 2022 (*n =* 40).

Among forty norovirus-positive AGE cases in infants, seventy unique controls were selected, of which six were selected twice, and four controls later became AGE cases. The first norovirus cases were detected in October 2018, and the last were detected in January 2022. The highest norovirus incidence occurred between October and December 2018 and in June 2019, coinciding with the rainy season in Nicaragua (Figure 1).

Evaluable genotype information was available for 30 of the 40 norovirus-positive AGE cases, including 21 (70%) GII.4 and nine (30%) non-GII.4 (GI.3 [*n* = 3], GI.5 [*n* = 2], GII.12 [*n* = 2], GII.14 [*n* = 1], and GII.17 [*n* = 1] (Table 1)). GII.4 cases tended to have higher median severity scores compared with non-GII.4. Among the 30 typeable cases, nine (30%) presented with fever, 12 (40%) received treatment in a public health clinic, four (13%) presented to the emergency department (ED), and three (10%) were hospitalized. GII.4 cases were more likely to receive treatment in a health care facility, including 83% of typeable norovirus cases seen in a clinic (10 of 12), and 100% of ED (four of four) and hospitalized (three of three) cases (Table 1). Two hospitalized GII.4 cases (67%) received intravenous rehydration.

In bivariate analysis of biologic, socioeconomic, environmental, and nutritional risk factors for norovirus, we found that male sex, a dirt floor in the household, lower length-for-age Z score, sharing cups or bottles with others, and recent contact with individuals with AGE symptoms had significant associations with norovirus-positive AGE cases (*p* < 0.1) (Table 2). In multivariable conditional logistic regression analysis adjusting for these five risk factors, many of the associations were attenuated or lost precision in the 95% confidence intervals. However, female sex remained protective against norovirus, as did higher LAZ score. A dirt floor in the home, sharing cups or bottles, and contact with individuals with AGE symptoms tended to confer excess risk for norovirus, with contact conferring the greatest risk (aOR: 40.28), though the estimates were imprecise (Table 3).

## 4. Discussion

In this population-based study of young Nicaraguan children, we confirmed some previously reported risk factors for norovirus AGE (i.e., dirt flooring in the household, low LAZ, contact with sick persons), and identified new risk factors (i.e., male sex, sharing drinks). For example, having contact with another person with diarrhea and/or vomiting in the past week, including household members and individuals outside the home, was the strongest risk factor for norovirus AGE in our study, as determined previously [8,9,11]. Additionally, the association of norovirus AGE and living in a household with dirt floors was not unexpected, as norovirus particles have been shown to persist on floors as dust [18,19], and it is conceivable that porous dirt floors would be harder to disinfect than tile or other hard-surface flooring. A prior study in eight LMICs showed that improved flooring as well as higher LAZ was protective against norovirus infection in the first two years of life [12]. Our study did not collect extensive data on, and thus did not identify nutrition practices that were associated with norovirus; this could be the focus of future studies that are designed to accurately capture a diverse range of food and beverage consumption among children at risk for norovirus.

We did not expect to find that female sex was substantially protective against norovirus AGE in this setting, with an adjusted odds ratio (OR) of 0.32 (0.12–0.89). An analysis of a large surveillance database in Germany also found that norovirus is more common in young boys versus girls [20]. It is not known whether there are inherent differences in susceptibility to norovirus infection in male versus female children due to biological factors, or whether there may be differences in behaviors allowed by caregivers based on a child’s sex. Increased risk of infectious diseases in males may be due to differences in the ratio of symptomatic to asymptomatic infection between sexes [21]. Further research is warranted to determine if child activities are associated with norovirus risk, and could potentially be modified to decrease norovirus burden. In addition, having a lower length-for-age Z-score was associated with norovirus AGE, which may reflect a weaker immune system in children with sub-optimal nutrition, or conversely, poor growth due to repeated past exposures to enteric pathogens.

Interestingly, we found that sharing a cup or bottle with another individual in the past week was associated with 3.24 (1.00, 10.48) times the odds of norovirus AGE as compared with children who did not share a cup or bottle, which had not previously been reported in the published literature to our knowledge. This finding supports a recent report that, in a mouse model, norovirus can replicate in salivary glands and infect through saliva [22]. In humans, PCR detection of norovirus in the saliva of infected individuals has been associated with recent vomiting [23] and higher viral load in feces [24]. This finding of potential oral–oral transmission is important when considering measures to prevent norovirus spread.

Finally, to inform the choice of genotypes to include in norovirus vaccine candidates, we compared clinical symptoms between children experiencing infection with the GII.4 genotype vs. other norovirus genotypes. All measures of severity (duration of the AGE episode, maximum number of stools in a 24 h period, duration of vomiting, presence of fever, health care seeking, and hospital admission) were generally higher among those who experienced a GII.4 norovirus AGE episode as compared with infection with other norovirus genotypes. These findings agree with prior studies conducted in high-income countries that included larger numbers of children, showing that GII.4 norovirus AGE episodes are associated with greater clinical severity than those due to non-GII.4 noroviruses [25,26,27].

Some limitations of this study include the relatively small number of cases, particularly when stratifying by norovirus genotype, which limited statistical power to precisely detect norovirus risk factors. In addition, the numbers of untypeable noroviruses (23%) precluded statistical testing of differences in symptom severity between norovirus genotypes. However, we did identify strong tendencies in the directions of association for protective and risk factors for norovirus, which are also biologically plausible. In addition, our findings on genotype-specific severity concur with previous reports. This study was conducted at a single geographic site, and the findings may not translate to other settings with different environmental, cultural, and socioeconomic features. However, several of our findings concurred with those from previous studies, suggesting that some norovirus risk factors, such as prior contact with norovirus-infected persons, are applicable to various settings. Seasonal patterns of norovirus infection have been observed in the study setting, with excess cases occurring during periods of heavy rain (generally May through October). No norovirus cases were detected in the calendar year 2020, likely because children from the primary cohort had aged out of the risk period for pediatric norovirus infection, and the secondary cohort had not been recruited yet. Modified AGE surveillance continued during the COVID-19 pandemic, with AGE episodes being reported during weekly phone calls and stool samples being collected during contactless pickups; nearly 100% of stools samples were collected for reported AGE episodes. In addition, the COVID-19 pandemic was unlikely to have reduced norovirus transmission rates, as physical distancing measures and school closures were not widely enforced in the study area.

## 5. Conclusions

Continued promotion of hygienic and physical distancing practices, particularly when a close contact is known to be symptomatic, are essential components of infant norovirus prevention in the absence of prophylactic vaccines. Furthermore, though a variety of circulating norovirus genotypes were identified in our study, our work implicates GII.4 as a target of future norovirus vaccines, as it was accompanied by more severe clinical symptoms.

## Figures and Tables

**Table 1:** Clinical characteristics of norovirus gastroenteritis episodes by genotype in a birth cohort of children in León, Nicaragua.

	N (%) or Median (IQR)			
Clinical Characteristic	Typeable Norovirus (*n* = 30)	GII.4 (*n* = 21)	Non-GII.4 (*n* = 9) ^a^	Untypeable Norovirus (*n* = 10)
Diarrhea				
Median duration in days (IQR)	2 (2, 3)	2.5 (2.0, 6.5)	2 (2, 2)	1 (1, 1)
Median number of stools in a 24 h period (IQR)	5 (4, 6)	6 (5, 7)	5 (4, 5)	4 (3, 5)
Vomiting				
Median duration in days	2 (1, 2)	2 (1, 2)	0.5 (0, 1)	-
Fever	9 (30.0)	8 (38.1)	1 (11.1)	2 (20.0)
Median gastroenteritis severity score (scale 0–15) ^b^ (IQR)	2.5 (1, 5)	4 (2, 5)	1 (1, 2)	1 (0, 2)
Received care at primary care clinic	12 (40.0)	10 (47.6)	2 (22.2)	1 (10.0)
Received care at emergency department	4 (13.3)	4 (19.0)	0	0
Admitted to hospital	3 (10.0)	3 (14.3)	0	0
Received zinc	8 (26.7)	5 (23.8)	3 (33.3)	2 (20.0)
Received intravenous fluid	3 (10.0)	3 (14.3)	0	0

Abbreviations: IQR = interquartile range. ^a^ Typeable non-GII.4 noroviruses include GI.3 (*n* = 3), GI.5 (*n* = 2), GII.12 (*n* = 2), GII.14 (*n* = 1), and GII.17 (*n* = 1). ^b^ Severity score calculated based on duration of diarrhea and or vomiting symptoms, the maximum number of stools reported per day, presence of fever, and receipt of intravenous fluid for dehydration. Adapted from the scale developed by Lee et al. [17].

**Table 2:** Characteristics of norovirus cases relative to age-matched controls in a birth cohort of children in León, Nicaragua ^a^.

Characteristic	Cases (*n* = 40)	Controls (*n* = 80)	*p*
*n* (%) or Mean (STD)			
Mean age at the time of norovirus case (in months)	12.1 (5.7)	11.6 (6.0)	0.7
**Birth Characteristics**			
Sex (% female)	12 (30.0)	29 (48.8)	0.05
Mode of delivery (% vaginal delivery)	19 (47.5)	45 (56.3)	0.4
Gestational age at birth (in completed weeks)	38.5 (1.4)	38.8 (1.0)	0.1
Mean birthweight (in grams)	3075 (419)	3111 (420)	0.7
Mean age of mother (years) at child’s birth	24.9 (6.7)	24.6 (4.9)	0.8
**Socioeconomic Indicators**			
Maternal educational attainment *(Missing: 2 cases, 3 controls)*			0.7
Completed primary education or less	8 (21.0)	14 (18.2)	
Completed any secondary education	30 (79.0)	63 (81.8)	
Mother employed at time of child’s birth	12 (30.0)	14 (17.5)	0.1
Crowding index (>2.5 people/bedroom)	10 (25.0)	22 (27.5)	0.8
Floor type (% dirt floor)	19 (47.5)	25 (31.3)	0.08
**Household Sanitation**			
Water source (% municipal in home)	31 (77.5)	70 (87.5)	0.2
Sanitation type (% indoor toilet)	22 (55.0)	56 (70.0)	0.1
Always uses ≥1 effective means of water purification ^b^	2 (5.0)	6 (7.5)	0.6
Water source interruption in the past week	2 (5.0)	8 (10.0)	0.5
Animals in the home, any			
Dog	29 (72.5)	48 (60.0)	0.2
Cat	10 (25.0)	22 (27.5)	0.8
Chickens	9 (22.5)	18 (22.5)	2
Pig	4 (10.0)	4 (5.0)	0.4
Rabbit	2 (5.0)	2 (2.5)	0.6
Bird	3 (7.5)	3 (3.75)	0.4
Mice	5 (12.5)	6 (6.25)	0.3
Other, including horses and/or ducks	6 (15.0)	5 (6.25)	0.2
**Personal Hygiene**			
Mother practices handwashing at appropriate moments ^c^	30 (75.0)	52 (65.0)	0.3
Use of alcohol hand sanitizer in home (% mothers using at least sometimes)	12 (30.0)	24 (30.0)	1
**Nutrition**			
Child was breastfed the previous day	29 (72.5)	56 (70.0)	0.8
Mean age of introduction of any supplementary foods/drink (in weeks)	3.2 (0.8)	3.3 (2.3)	0.5
Mean weight-for-age Z-score	0.2 (1.2)	0.5 (0.9)	0.2
Mean length-for-age Z-score	−0.095 (1.3)	0.38 (1.2)	0.05
Mean BMI-for-age Z score	0.35 (1.6)	0.37 (1.2)	0.9
Ate uncooked fruit/vegetable in the past week	32 (80.0)	60 (75.0)	0.5
Ate seafood in the past week	3 (7.5)	16 (20.0)	0.1
Ate outside the home in the past week	19 (47.5)	26 (32.5)	0.1
Shared a bottle or cup with another person in the past week	24 (60.0)	28 (35.0)	0.01
**Interpersonal Contact**			
Other child in home in diapers	6 (15.0)	11 (13.8)	0.9
Attended a social event in the past week	12 (30.0)	15 (18.8)	0.2
Floor type (% dirt floor)	12 (30.0)	27 (35.5)	0.6
Used public transportation in the past week	14 (35.0)	31 (38.8)	0.7
Went swimming in the past week	1 (2.5)	3 (3.8)	1
Had contact with anyone with diarrhea and/or vomiting in the past week	7 (17.5)	3 (3.75)	0.02
**Gastroenteritis risk factors**			
Prior episode of gastroenteritis (of any cause)	29 (72.5)	49 (61.3)	0.2

^a^ Cases were matched 1:2 with controls ± 3 months of age. Infants with a history of norovirus gastroenteritis by RT-qPCR were not eligible to be controls. Controls who were selected more than once were included multiple times in the predictors analysis, and controls who later became cases were included in both case and control columns. *p*-values have not been adjusted for multiple comparisons, or for individuals who contributed data multiple times. ^b^ Water treatment options included: using a water filter (including sand and mud/ceramic filters); boiling water; adding bleach or chlorine; solar disinfection; straining through a cloth; letting water settle; purchasing purified water. ^c^ Appropriate moments included: after caring for a sick person; before eating; before preparing food; after using the bathroom; after changing diapers.

**Table 3:** Associations between selected variables and norovirus infection in a nested case–control study of children in León, Nicaragua.

	Crude OR (95% CI)	Adjusted OR (95% CI) ^a^
Sex (female vs. male)	0.43 (0.19, 1.0)	0.32 (0.12, 0.89)
Floor type (dirt floor vs. solid floor)	2.15 (0.94, 4.93)	2.6 (0.96, 6.82)
Mean length-for-age Z-score	0.74 (0.54, 1.01)	0.66 (0.44, 0.97)
Shared a bottle or cup with another person in the past week	3.59 (1.39, 9.29)	3.24 (1.00, 10.48)
Had contact with anyone with diarrhea and/or vomiting in the past week	11.13 (1.34, 92.45)	40.28 (1.00, ∞)

^a^ Model adjusted for all other variables in the column and residual age difference between cases and controls. Bivariate predictors at *p* < 0.1 from Table 2 were included in the adjusted model.

## Data Availability

The data are not publicly available due to the inclusion of protected health information, including names, addresses, and dates of birth. De-identified data presented in this study are available on request from the corresponding author.
